# Optimisation of the differing conditions required for bone formation *in vitro* by primary osteoblasts from mice and rats

**DOI:** 10.3892/ijmm.2014.1926

**Published:** 2014-09-08

**Authors:** ISABEL R. ORRISS, MARK O.R. HAJJAWI, CARMEN HUESA, VICKY E. MACRAE, TIMOTHY R. ARNETT

**Affiliations:** 1 Department of Cell and Developmental Biology, University College London, London, UK; 2 Department of Comparative Biomedical Sciences, Royal Veterinary College, London, UK; 3 The Roslin Institute and Royal (Dick) School of Veterinary Studies, University of Edinburgh, Edinburgh, UK

**Keywords:** osteoblast, bone formation, alkaline phosphatase, mineralisation

## Abstract

The *in vitro* culture of calvarial osteoblasts from neonatal rodents remains an important method for studying the regulation of bone formation. The widespread use of transgenic mice has created a particular need for a reliable, simple method that allows the differentiation and bone-forming activity of murine osteoblasts to be studied. In the present study, we established such a method and identified key differences in optimal culture conditions between mouse and rat osteoblasts. Cells isolated from neonatal rodent calvariae by collagenase digestion were cultured for 14–28 days before staining for tissue non-specific alkaline phosphatase (TNAP) and bone mineralisation (alizarin red). The reliable differentiation of mouse osteoblasts, resulting in abundant TNAP expression and the formation of mineralised ‘trabecular-shaped’ bone nodules, occurred only following culture in α minimum essential medium (αMEM) and took 21–28 days. Dexamethasone (10 nM) inhibited bone mineralisation in the mouse osteoblasts. By contrast, TNAP expression and bone formation by rat osteoblasts were observed following culture in both αMEM and Dulbecco’s modified Eagle’s medium (DMEM) after approximately 14 days (although ~3-fold more effectively in αMEM) and was strongly dependent on dexamethasone. Both the mouse and rat osteoblasts required ascorbate (50 μg/ml) for osteogenic differentiation and β-glycerophosphate (2 mM) for mineralisation. The rat and mouse osteoblasts showed similar sensitivity to the well-established inhibitors of mineralisation, inorganic pyrophosphate (PP_i_) and adenosine triphosphate (ATP; 1–100 μM). The high efficiency of osteogenic differentiation observed following culture in αMEM, compared with culture in DMEM possibly reflects the richer formulation of the former. These findings offer a reliable technique for inducing mouse osteoblasts to form bone *in vitro* and a more effective method for culturing bone-forming rat osteoblasts.

## Introduction

Osteoblasts, the cells responsible for bone formation, are derived from mesenchymal stem cells. The *in vitro* culture of osteoblasts constitutes a central part of research into the regulation of bone cell function. A number of different approaches have been developed to study osteoblasts *in vitro*, including primary cell cultures, immortalised osteoblast-like cell lines and bone organ cultures. In combination, these methods have provided abundant information as to the regulation of osteoblast proliferation, differentiation, survival and function.

A number of different techniques for obtaining primary osteoblasts have been described (1–4). These methods use cells isolated from a number of different skeletal locations (e.g., long bones, calvariae) and animal models (e.g., human, rat, mouse). Of these, the *in vitro* culture of calvarial osteoblasts from neonatal rodents remains a main method for studying the regulation of osteoblast function. The widespread use of transgenics has created a particular need for a reliable, simple method that allows the differentiation and bone-forming activity of mouse osteoblasts to be investigated directly.

Rat primary bone cell cultures were first described in 1964 by Peck *et al* (5), who isolated cells from the parietal and frontal bones of fetal and neonatal calvariae using collagenase digestion. The isolated cells proliferated *in vitro* and exhibited high tissue non-specific alkaline phosphatase (TNAP) activity; however, the cultures were contaminated with other cell types, such as fibroblasts. In 1974, Wong and Cohn used sequential collagenase digestion to obtain a more homogenous population of osteoblasts (6). The first description of the formation of bone nodules by differentiating osteoblasts released enzymically from calvarial bone and cultured with β-glycerophosphate ascorbate and dexamethasone was by Bellows *et al* in 1986 (7).

The calvarial osteoblast bone formation assay has a number of advantages. Firstly, it allows the key function of osteoblasts, namely bone formation, to be studied quantitatively (1). Secondly, it enables the processes of bone matrix deposition and mineralisation to be studied separately (8). Thirdly, osteoblast activity can be studied in an environment that is relatively free from the influence of other cell types normally found in bone, such as endothelial and haematopoietic cells. Fourthly, it allows the extracellular environment to be tightly controlled (e.g., pH, pO_2_) in a manner which is not possible *in vivo* or using bone organ cultures (9,10). Lastly, osteoblasts can be studied at clearly identified stages of differentiation from the immature, proliferating cells present early in the cultures through to the mature bone-forming osteoblasts in late-stage cultures.

There are now numerous reported methods for isolating and culturing rodent calvarial osteoblasts. The objectives of this study were to: i) establish clear, simple methods for culturing mouse and rat osteoblasts *in vitro*; and ii) identify the key differences between the protocols which need to be taken into account to ensure successful osteoblast cultures.

## Materials and methods

### Reagents

All tissue culture reagents, including Dulbecco’s modified Eagle’s medium (DMEM) (no. 11880), α Minimum essential medium (αMEM) (no. 22571) and fetal calf serum (FCS) (no. 102701) were purchased from Invitrogen Life Technologies (Paisley, UK); unless otherwise mentioned, other reagents were obtained from Sigma-Aldrich (Dorset, UK).

### Rat calvarial isolation [trypsin-collagenase-collagenase (TCC) method]

Primary rat osteoblasts were obtained by sequential enzyme digestion of excised calvarial bone from 2-day-old neonatal Sprague-Dawley rats using a 3-step process [(TCC); 1% trypsin in PBS for 10 min; 0.2% collagenase type II in Hank’s balanced salt solution (HBSS) for 30 min; 0.2% collagenase type II in HBSS for 60 min]. The first 2 digests were discarded and the cells from the final digest were resuspended in DMEM supplemented with 10% FCS, 2 mM L-glutamine, 5% gentimicin, 100 U/ml penicillin, 100 μg/ml streptomycin and 0.25 μg/ml amphotericin. The cells were cultured for 2–4 days in a humidified atmosphere of 5% CO_2_-95% air at 37°C in 75 cm^2^ flasks until confluent (1 calvarial bone/flask). All institutional and national guidelines for the care and use of laboratory animals were followed.

### Mouse calvarial isolation [collagenase-collagenase-EDTA-collagenase (CCEC) method]

Primary mouse osteoblasts were obtained by sequential enzyme digestion of excised calvarial bone from 2- to 4-day-old neonatal mice (129/sv) using a 4-step process (CCEC). The first digest (1 mg/ml collagenase type II in HBSS for 10 min) was discarded. The following 3 digests (fraction 1, 1 mg/ml collagenase type II in HBSS for 30 min; fraction 2, 4 mM EDTA in PBS for 10 min; fraction 3, 1 mg/ml collagenase type II in HBSS for 30 min) were retained. During the final digestion, the cells obtained from fractions 1 and 2 were resuspended in αMEM supplemented with 10% heat-inactivated FCS (HI FCS), 5% gentamicin, 100 U/ml penicillin, 100 μg/ml streptomycin and 0.25 μg/ml amphotericin. The cells from fraction 3 were then combined with fractions 1 and 2 for expansion. The cells were cultured in 25 cm^2^ flasks (1 calvariae/flask) or 75 cm^2^ flasks (3 calvaria/flask) for 4–5 days in a humidified atmosphere of 5% CO_2_-95% air at 37°C until confluent. To heat inactivate, FCS was treated at 55°C for 1 h. The above 2 protocols were each tested on both rat and mouse calvarial bone.

### Osteoblast culture

Upon confluence, the cells were plated into 24-, 12- or 6-well trays (Falcon^®^, BD Biosciences, Franklin Lakes, NJ, USA) at 2.5×10^4^, 5×10^4^ and 10^5^ cells/well, respectively. To determine the optimal tissue culture medium for growing rodent osteoblasts, the cells were cultured in both DMEM and αMEM for up to 28 days. In contrast to DMEM, αMEM has a richer formulation, containing both essential and non-essential amino acids (including proline), nucleosides and ascorbate. DMEM (no. 11880), αMEM (no. 22571), FCS (batch tested: no. 102701) were obtained from Invitrogen Life Technologies. The effects of FCS heat inactivation (1 h at 55°C), dexamethasone (10 nM) and β-glycerophosphate (0–10 mM) on the ability of the cells to form mature, bone-forming osteoblasts were also examined.

All tissue culture media (i.e., including αMEM) were supplemented with 50 μg/ml ascorbate and the medium was half changed every 3 days. Medium pH, pCO_2_ and pO_2_ were monitored throughout the experiment using a blood gas analyser (ABL-705; Radiometer, Crawley, UK). The experiments were carefully pH-controlled as osteoblast TNAP activity and bone mineralisation are extremely sensitive to inhibition by acidosis (9).

The responses of rat and mouse calvarial osteoblasts to the well-known inhibitors of bone mineralisation, inorganic pyrophosphate (PP_i_) and adenosine triphosphate (ATP) (8,11) were also compared in 14–28-day cultures.

### Fixation, staining and analysis

The experiments were terminated by washing cell layers with PBS prior to fixation in 2.5% glutaraldehyde for 5 min. The cell layers were then washed twice with distilled water (dH_2_O) and left to air dry prior to staining. Mineralised nodules were stained with 2% alizarin red S (w/v), pH 4.5 in dH_2_O for 30 min followed by 3 washes with dH_2_O. The TNAP staining of osteoblast cell layers (~30 min in the dark, followed by a wash with dH_2_O) was carried out using a commercially available kit (Sigma-Aldrich). All plates were allowed to air dry before scanning for analysis. Mineralised bone nodule formation and TNAP expression were quantified by image analysis, as previously described (1).

### Statistical analysis

Statistical comparisons (InStat; GraphPad Software, Inc., La Jolla, CA, USA) were made using both parametric (one-way analysis of variance and adjusted using the Bonferroni method) and non-parametric Kruskall-Wallis and adjusted using the Dunn method tests. These methods yielded similar results (all figures where statistical significance is shown). Representative data are presented as the means ± SEM for 6 replicates. Results presented are for representative experiments that were each repeated at least 3 times.

## Results

### Bone formation by rat calvarial osteoblasts: effects of culture medium, dexamethasone and β-glycerophosphate

**Figure 1:**
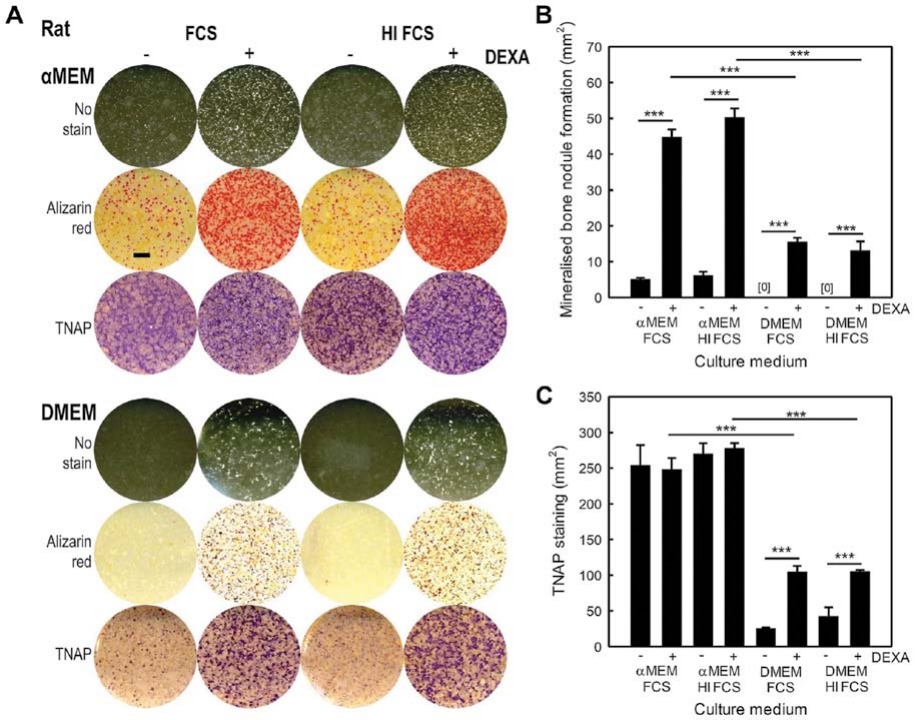
Rat calvarial osteoblast cultures form bone more abundantly in αMEM. All cells were cultured in 50 μg/ml ascorbate and 2 mM β-glycerophosphate. (A) The images are representative, whole well reflective light scans of rat osteoblast cell layers cultured using DMEM or αMEM supplemented with 10% fetal calf serum (FCS) or heat-inactivated FCS (HI FCS), with or without 10 nM dexamethasone. Cell layers are either unstained (white), stained with alizarin red to show bone mineralisation (red) or for tissue non-specific alkaline phosphatase (TNAP) expression (purple). Scale bar, 5 mm. (B) The level of bone mineralisation was 3-fold higher in the cells cultured in αMEM compared to DMEM. In cultures without dexamethasone, bone mineralisation was 85% lower (αMEM) or completely absent (DMEM). Heat inactivation of FCS did not have an effect on the level of bone formation. (C) Levels of TNAP expression were 2.5-fold higher in the cells cultured in αMEM. The absence of dexamethasone had no effect on TNAP expression when the cells were grown in αMEM, but TNAP expression was reduced by 3- to 4-fold in the cells cultured in DMEM. Heat inactivation of FCS did not affect TNAP expression. Values are the means ± SEM (n=6 replicate wells), ^***^p<0.001.

Osteoblasts isolated from rat calvariae formed ‘trabecular-shaped’ mineralised bone nodules that were clearly visible by the eye, and expressed significant amounts of TNAP when cultured in both DMEM and αMEM. However, bone formation and TNAP expression were 2- to 3-fold higher in the cells cultured in αMEM than in the cells cultured in DMEM (Fig. 1A–C). The onset of mineralisation in the DMEM cultures occurred at day 10, with optimal bone formation at days 14–17, whereas the αMEM cultures showed marked acceleration, with mineralisation commencing at day 7 and striking bone formation observed between days 10–14. The heat inactivation of the FCS added to the culture media had no effect on mineralised bone nodule formation and TNAP staining.

Rat osteoblasts cultured in DMEM without dexamethasone failed to form any bone and showed 3- to 4-fold lower TNAP expression (Fig. 1B and C). However, when the cells were cultured in αMEM without dexamethasone, some mineralised nodule formation was evident, albeit at greatly reduced (−85%) levels, compared to the cells cultured with dexamethasone (Fig. 1B). TNAP expression was unaltered by dexamethasone in the cells cultured in αMEM (Fig. 1C).

**Figure 2:**
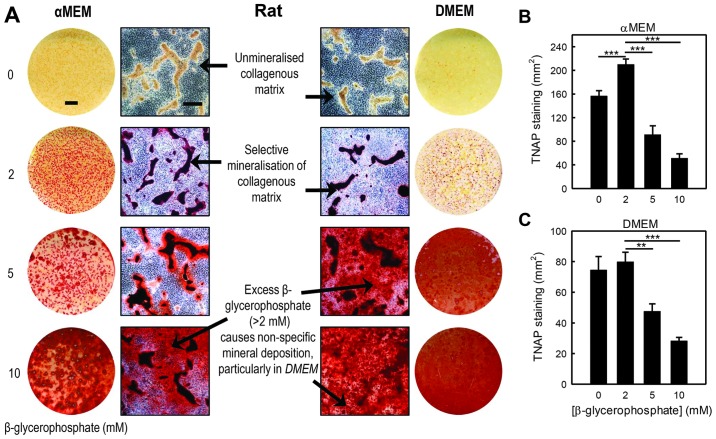
Excess β-glycerophosphate causes non-specific mineralisation and decreases tissue non-specific alkaline phosphatase (TNAP) expression in rat calvarial osteoblast cultures. All cells were cultured with heat-inactivated FCS (HI FCS), 50 μg/ml ascorbate and 10 mM dexamethasone. (A) Low power whole well scans and higher resolution phase contrast microscopy images showing alizarin red stained rat osteoblast cell layers cultured in DMEM or αMEM with 0–10 mM β-glycerophosphate. Without β-glycerophosphate mineralisation did not occur, whilst 2 mM β-glycerophosphate resulted in the selective mineralisation of the collagenous matrix. Culture with ≥5 mM β-glycerophosphate resulted in non-specific dystrophic mineralisation, the levels of which were higher in the cells cultured in DMEM. Scale bars: whole well, 5mm; phase contrast micrographs, 500 μm. (B) In the cells cultured in αMEM, 5 and 10 mM β-glycerophosphate reduced TNAP expression by 55 and 70%, respectively. (C) In DMEM, TNAP levels were decreased by 0 and 65% at 5 and 10 mM β-glycerophosphate, respectively. Values are the means ± SEM (n=6 replicate wells), ^**^p<0.01, ^***^p<0.001.

β-glycerophosphate, which is hydrolysed by TNAP, is widely used in osteoblast cultures as an inorganic phosphate (P_i_) source for mineralisation. The concentrations typically used vary from 2–10 mM. However, an excessive concentration of β-glycerophosphate (≥5 mM) has been shown to cause widespread, non-specific (‘dystrophic’) mineralisation, a phenomenon that differs greatly from true bone formation and is associated with reduced cell viability (1). We found that in the cells cultured in both DMEM and αMEM, mineralisation failed to occur in the absence of β-glycerophosphate (Fig. 2A). In the presence of 2 mM β-glycerophosphate, selective mineralisation of collagenous trabecular structures occurred in the cells cultured in both media, evidenced by intense alizarin red staining. At a concentration of 5 mM, β-glycerophosphate caused the abundant, non-specific deposition of mineral in the DMEM cells cultures but not in the αMEM cell cultures, perhaps reflecting the greater cellular activity in the latter medium. The widespread, non-specific mineralisation of osteoblast cell layers was observed in both αMEM and DMEM cell cultures containing 10 mM β-glycerophosphate (Fig. 2A). Increasing the β-glycerophosphate concentration above 2 mM also had a marked inhibitory effect on the amount of TNAP staining observed in the rat osteoblast cell layers, with reductions of up to 3-fold observed (Fig. 2B and C).

### Bone formation by mouse calvarial osteoblasts: effects of culture medium, dexamethasone and β-glycerophosphate, and differences from rat osteoblasts

**Figure 3:**
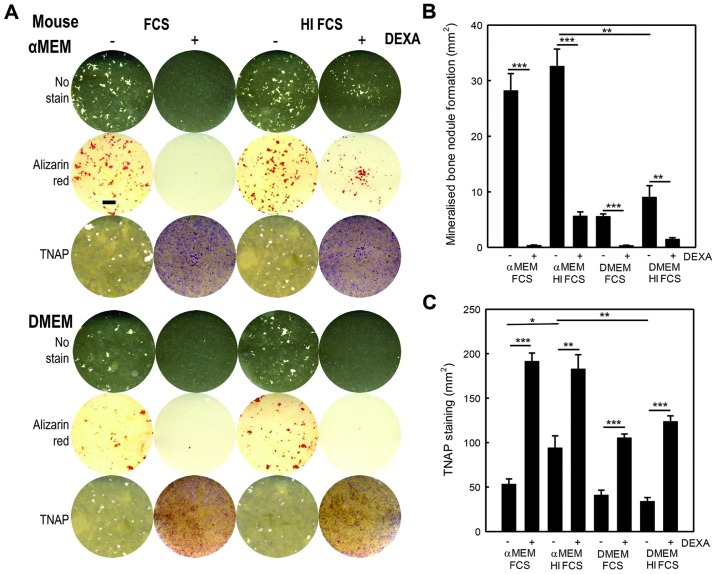
Mouse calvarial osteoblasts require different culture conditions in order to form mineralised bone nodules *in vitro* from rat osteoblasts. All cells were cultured in 50 μg/ml ascorbate and 2 mM β-glycerophosphate. (A) The images are representative whole well reflective light scans of mouse osteoblast cell layers cultured using DMEM or αMEM supplemented with 10% fetal calf serum (FCS) or heat-inactivated FCS (HI FCS), with or without 10 nM dexamethasone. Cell layers are either unstained or stained with alizarin red to show bone mineralisation (red) or for tissue non-specific alkaline phosphatase (TNAP) expression (purple). Scale bar, 5 mm. (B) Bone mineralisation was 4-fold higher when the cells were cultured in αMEM compared to DMEM. In cultures with dexamethasone, mineralisation was >80% lower. Using HI FCS resulted in a slight, non-significant increase in bone formation. (C) TNAP expression was ~50% higher in the cells cultured in αMEM. The absence of dexamethasone increased TNAP expression by up to 3-fold in cells cultured in both αMEM and DMEM. Using HI FCS did not affect TNAP expression. Values are the means ± SEM (n=6 replicate wells), ^*^p<0.05, ^**^p<0.01, ^***^p<0.001.

Osteoblasts from mouse calvariae also grew much more successfully in αMEM, such that bone formation was at least 3-fold higher than in the cells cultured in DMEM (Fig. 3A and B). The tissue culture medium also affected TNAP, the expression of which was up to 2-fold higher in the cells cultuerd in αMEM compared with the cells cultured in DMEM (Fig. 3A and C). There was a modest trend towards increased bone formation in the cell cultures supplemented with HI FCS, compared with untreated FCS, although TNAP expression was not affected (Fig. 3B and C).

In contrast to the response of rat osteoblasts, the addition of 10 nM dexamethasone to the cell cultures reduced mineralised bone formation by >90% (Fig. 3B). Surprisingly, TNAP expression was up to 3-fold higher in the cells treated with dexamethasone compared to the untreated cells.

**Figure 4:**
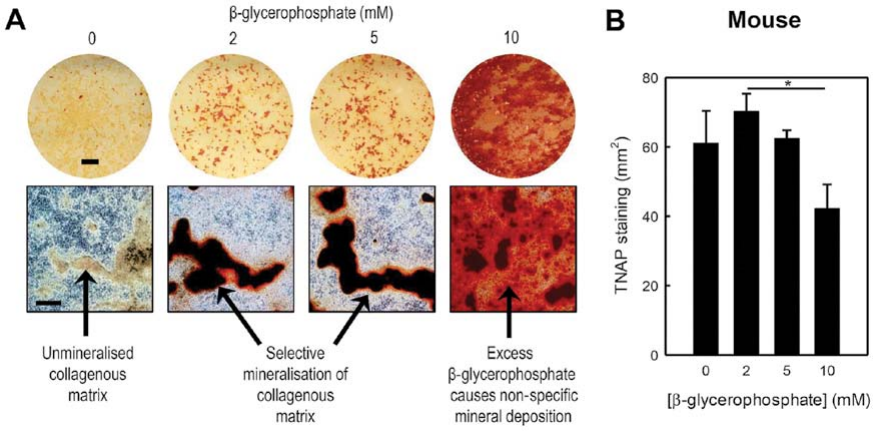
Excess β-glycerophosphate causes non-specific mineralisation and decreases tissue non-specific alkaline phosphatase (TNAP) expression in mouse calvarial osteoblast cultures. All cells were cultured in αMEM with heat-inactivated FCS (HI FCS) and 50 μg/ml ascorbate. (A) Low power whole well scans and higher resolution phase contrast microscopy images showing alizarin red stained mouse osteoblast cell layers cultured in αMEM with 0–10 mM β-glycerophosphate. In the absence of β-glycerophosphate mineralisation does not occur, whilst 2–5 mM β-glycerophosphate results in the selective mineralisation of the collagenous matrix. The 10 mM β-glycerophosphate results in widespread, non-specific dystrophic mineralisation of cell layers. Scale bars: whole well, 5 mm; phase contrast micrographs, 500 μm. (B) The 10 mM β-glycerophosphate reduced TNAP expression by 40%. Values are the means ± SEM (n=6 replicate wells), ^*^p<0.05.

The effects of β-glycerophosphate on the mineralisation of mouse calvarial osteoblasts cultured in αMEM were broadly similar to those observed for rat osteoblasts. No mineralisation was observed in the absence of β-glycerophosphate; normal, selective mineralisation of the collagenous matrix occurred in the presence of 2–5 mM β-glycerophosphate, whereas 10 mM β-glycerophosphate caused non-specific, dystrophic mineralisation and a significant inhibition of TNAP staining (Fig. 4A and B).

**Figure 5:**
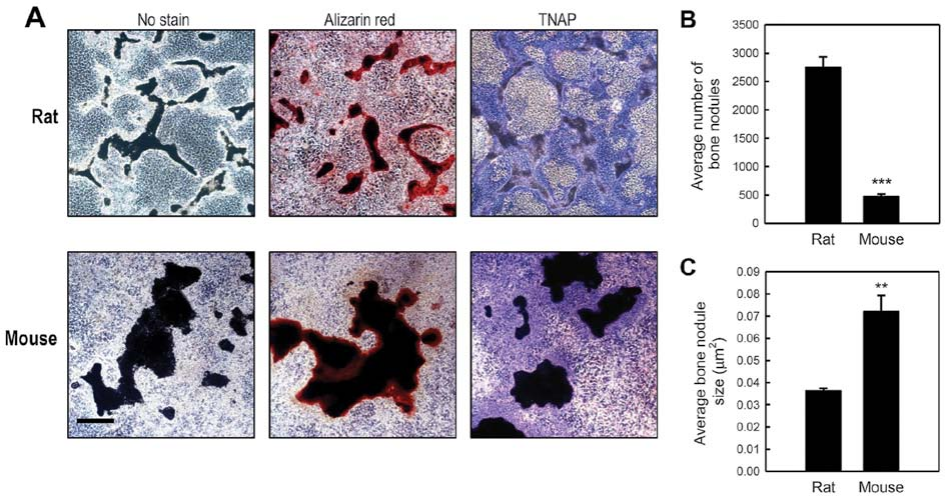
The mineralised bone nodules formed by rat osteoblasts are smaller but more abundant than mouse osteoblasts. All cells were cultured with heat-inactivated FCS (HI FCS) and 50 μg/ml ascorbate and 2 mM β-glycerophosphate. (A) Phase contrast microscopy images highlight the significant differences in the size and number of bone nodules formed by rodent osteoblasts. Images show unstained (white), alizarin red (red) and tissue non-specific alkaline phosphatase (TNAP) (purple) stained bone nodules. Scale bar, 500 μm. (B) The number of mineralised bone nodules formed in cultures of rat osteoblasts was 6-fold higher than mouse osteoblasts. (C) The size of the bone nodules formed was 2-fold higher in mouse osteoblast cultures. Values are the means ± SEM (n=6 replicate wells), ^**^p<0.01, ^***^p<0.001.

Optimal bone formation by mouse osteoblasts required ~21–28 days, and was thus ~2-fold slower than for rat osteoblasts seeded at the same density. The individual bone structures formed by mouse osteoblasts were larger and more nodule-like than those formed by rat osteoblasts, although much less abundant (Fig. 5A–C). Mouse osteoblasts grew best in 6-well plates; in 12 or 24-well plates, marked peeling of the cell layers tended to occur before the onset of bone formation (data not shown). Rat osteoblasts, on the other hand, were cultured successfully in 6, 12 or 24-wells. Tissue culture plastics from several different manufacturers (BD Biosciences; Corning, Inc., Corning, NY, USA; Thermo Fisher Scientific, Inc., Waltham, MA, USA) were also tested: no significant differences in the level of bone formation were observed (data not shown).

### Comparison of osteoblast isolation protocols with or without trypsin

**Figure 6:**
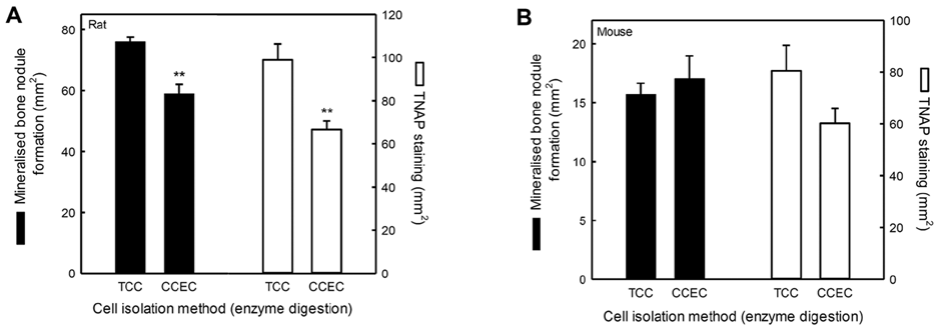
Tissue culture conditions are more important to the success of the culture than the initial method of obtaining cells. In order to determine how important the method of isolation is in the success of the culture, rodent cells were obtained using both the trypsin-collagenase-collagenase (TCC) and collagenase-collagenase-EDTA-collagenase (CCEC) digestion protocols. Cells were cultured in αMEM with heat inactivated FCS (HI FCS), 50 μg/ml ascorbate and 2 mM β-glycerophosphate (plus 10 nM dexamethasone, rat only) for up to 28 days. Mature, mineralising osteoblasts were generated from rat and mouse calvarial cells using both isolation methods. (A) The level of bone formation and tissue non-specific alkaline phosphatase (TNAP) expression were 25 and 35% lower, respectively, in rat cells obtained using CCEC digestion. (B) The method of isolation did not affect the level of bone mineralisation or TNAP expression in mouse osteoblasts. Values are the means ± SEM (n=6 replicate wells), ^**^p<0.01.

Rodent osteoblasts were isolated using both TCC and CCEC digestion protocols and cultured in αMEM with HI FCS, 50 μg/ml ascorbate and 2 mM β-glycerophosphate (plus 10 nM dexamethasone for rat cells). We found that mature, bone-forming osteoblasts that expressed TNAP were generated from rat and mouse calvarial cells using both methods, although the CCEC protocol appeared somewhat less efficient for rat cells (Fig. 6). The optimal conditions determined in this study for culturing bone-forming rodent osteoblasts are summarised in Table I.

### The inhibitors of mineralisation, PP_i_ and ATP, exert similar functional effects in cultures of rat and mouse osteoblasts

**Figure 7:**
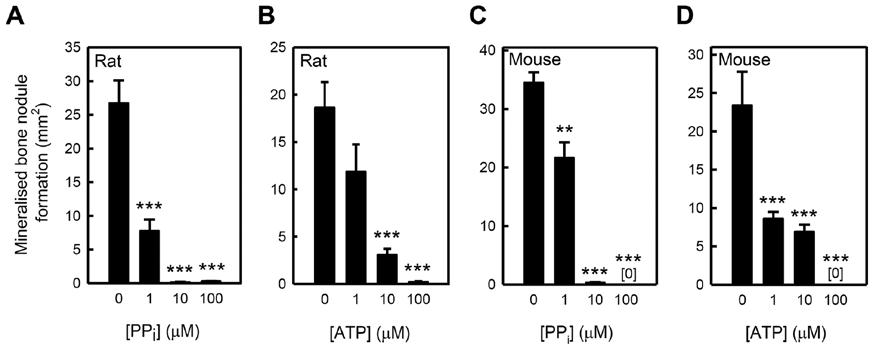
The inhibitors of mineralisation, inorganic pyrophosphate (PP_i_) and ATP, exert similar functional effects in cultures of rat and mouse osteoblasts. In rat osteoblasts, (A) PP_i_ and (B) adenosine triphosphate (ATP) inhibited mineralisation from 1 and 10 μM, respectively. At 100 μM mineralisation was decreased by >98%. In mouse osteoblasts, both (C) PP_i_ and (D) ATP blocked mineralisation from concentrations of 1 μM. At 100 μM, mineralisation was completely abolished. Values are the means ± SEM (n=6 replicate wells), ^**^p<0.01, ^***^p<0.001.

We compared the effects of the mineralisation inhibitors, PP_i_ and ATP, in rat and mouse calvarial osteoblast cultures. Similar dose-dependent responses were observed for both species. PP_i_ caused partial inhibition of mineralisation at a dose of 1 μM and near-total inhibition at concentrations ≥10 μM, whereas ATP had a more graded effect, with near-total inhibition observed only at 100 μM (Fig. 7).

## Discussion

The *in vitro* culture of rodent osteoblasts is a key research tool in bone biology. The increasingly widespread use of transgenic mouse models in research means that a reliable and effective method for culturing murine osteoblasts that results in the formation of discretely-mineralised bone nodules is required. This study determined the optimal conditions required for the successful culture of rodent osteoblasts and identified a number of key differences between rat and mouse cells which need to be taken into account for future study (Table I).

Significant differences in the behaviour of rat and mouse osteoblasts *in vitro* were observed. Rat osteoblast cultures were considerably shorter in duration typically being ~14 days, whilst mouse osteoblasts required a minimum of 21 days in order to form mineralised matrix nodules. There were also marked differences in the appearance of the bone that was formed; rat osteoblasts typically produced smaller bone nodules that had a ‘trabecular-shaped’ appearance. By contrast, in mouse osteoblast cultures, the bone nodules were larger but fewer in number.

This study compared the effects of two different culture media, a basic DMEM and a more nutrient-rich αMEM. The formulation of αMEM is designed to closely approximate the protein composition of cells (12) and contains a higher concentration of amino acids and nucleotides than DMEM. Abundant bone mineralisation and TNAP expression was evident when rat calvarial osteoblasts were cultured in both DMEM and αMEM. However, cells cultured in αMEM formed more bone, expressed higher levels of TNAP and began to mineralise more rapidly (after ~7 days in culture compared to ~10 days for DMEM). Thus, by culturing in αMEM, the efficiency of the cultures was increased and the overall duration of the experiment was reduced. This increased osteogenic activity of rat osteoblasts cultured in αMEM is most likely due to the richer formulation of the medium. It is noteworthy that proline and ascorbate, which are required for collagen synthesis, are both constituents of αMEM but not DMEM. By contrast, mouse osteoblasts showed lower osteogenic activity and significant bone formation was observed only in αMEM cultures. This suggests that mouse osteoblasts may require more nutritional support *in vitro* than rat osteoblasts. In addition to the experiments described in this study using osteoblasts from 129/sv mice (an inbred strain), we also successfully cultured bone-forming osteoblasts from C57BL/6 mice in αMEM, with no significant differences observed (data not shown).

FCS contains numerous growth factors and proteins which promote cell growth and survival and is a key supplement to tissue culture media. The heat inactivation of FCS is typically performed to degrade heat-labile components, such as complement (13), but it may also denature other proteins present in the serum. Our experiments indicated that the heat inactivation of serum had no detectable impact on either rat or mouse osteoblast cultures.

Dexamethasone was first shown to promote the differentiation of rat calvarial osteoblasts by Bellows *et al* in the 1980s (7). In agreement with their study, we found that rat bone cells cultured in DMEM required dexamethasone in order to form mineralised bone nodules and express significant amounts of TNAP. However, small amounts of bone formation did occur in the absence of dexamethasone when the rat cells were cultured in αMEM, reflecting the more strongly osteogenic properties of this medium. Surprisingly, TNAP expression by rat cells was unaffected by the presence or absence of dexamethasone in αMEM.

It has been previously demonstrated that dexamethasone inhibits proliferation and osteogenic differentiation in mouse osteoblast cultures (14,15). We also found that the addition of dexamethasone inhibited bone formation by mouse osteoblasts; this was associated with an increased TNAP expression after 28 days of culture. This observation may well be consistent with an inhibitory effect of dexamethasone on osteogenesis as TNAP expression has been reported to peak prior to the onset of bone formation in primary osteoblast cultures (8,16).

β-glycerophosphate, a widely used supplement in osteoblast tissue culture medium, is hydrolysed by TNAP to provide the P_i_ required for mineralisation. The concentrations typically used range between 2 and 10 mM. A previous study indicated that in rat osteoblast cultures, excessive amounts of β-glycerophosphate (≥5 mM) reduce cell viability and cause widespread, non-specific mineral deposition that differs greatly from true bone formation (1). The present study demonstrates that extensive dystrophic mineralisation also occurs when mouse osteoblasts are cultured with excessive amounts of β-glycerophosphate (10 mM). The higher tolerance of mouse osteoblasts to β-glycerophosphate may reflect differences in basal TNAP activity. In both rat and mouse osteoblasts, the concentrations of β-glycerophosphate that caused non-specific mineralisation also resulted in decreased TNAP expression. This may be due to the inhibition of the enzymatic activity of TNAP by P_i_ (17) or to the general detrimental effects of dystrophic mineralisation on cell viability (1).

We compared two different protocols for isolating osteoblasts from rodent calvariae. The first method, TCC, utilises only the cells from the final collagenase digestion (1). The second method, CCEC, retains and pools the cells from three of the four digestions (18). We found that both protocols were of broadly similar efficacy in generating mature, TNAP-positive, bone forming osteoblasts from both rat and mouse calvariae. Thus, it appears that the tissue culture conditions (i.e., media and supplements) are more important for osteogenesis than the initial method of obtaining cells.

PP_i_ and ATP are well-known inhibitors of bone mineralisation (8,11). In the present study, we demonstrate that low micromolar concentrations of PP_i_ and ATP block the mineralisation of the collagenous matrix in cultures of both rat and mouse osteoblasts; ATP was approximately an order of magnitude more potent in mouse osteoblasts. ATP inhibits bone mineralisation by two distinct mechanisms: signalling via P2 receptors and through the breakdown of ATP by NPP1 to produce PP_i_ (8). Thus, the increased potency of ATP in mouse osteoblasts may reflect a higher expression of P2 receptors or of NPP1.

The results presented herein, based on simple methods, should prove useful not only for laboratories wishing to establish osteogenic cultures of mouse osteoblasts, but also for those requiring a more effective technique for culturing bone-forming rat osteoblasts.

## Figures and Tables

**Table I:** Summary of the optimal conditions for culturing bone-forming rodent osteoblasts.

Conditions	Rat	Mouse
Cell isolation protocol	TCC	TCC or CCEC
Cell yield/calvariae (after expansion for 2–3 days)	7×10^6^–10^7^	3–5×10^6^
Basal medium	DMEM or αMEM Cultures 3–4 days shorter in αMEM	αMEM
FCS	10% FCS or HI FCS	10% FCS or HI-FCS
Dexamethasone	10 nM	Not required
β-glycerophosphate	2 mM	2–5mM
Ascorbate	50 μg/ml	50 μg/ml
Plate format	6-, 12- or 24-well plates	6-well plates
Culture duration	14–17 days in DMEM 10–14 days in αMEM	21–28 days

TCC, trypsin-collagenase-collagenase; CCEC, collagenase-collagenase-EDTA-collagenase.
